# Bladder preservation in non-metastatic muscle-invasive bladder cancer (MIBC): a single-institution experience

**DOI:** 10.3332/ecancer.2016.657

**Published:** 2016-07-14

**Authors:** Marianna A. Gerardi, Barbara A. Jereczek-Fossa, Dario Zerini, Alessia Surgo, Samantha Dicuonzo, Ruggero Spoto, Cristiana Fodor, Elena Verri, Maria Cossu Rocca, Franco Nolè, Matteo Muto, Matteo Ferro, Gennaro Musi, Danilo Bottero, Deliu V. Matei, Ottavio De Cobelli, Roberto Orecchia

**Affiliations:** 1 Department of Oncology and Haemato-oncology, University of Milan, 20122 Milan, Italy; 2 Division of Radiotherapy, European Institute of Oncology IRCCS, 20141 Milan, Italy; 3 Medical Division of Urogenital Tumours, European Institute of Oncology IRCCS, 20141 Milan, Italy; 4 Division of Urologic Cancer Surgery, European Institute of Oncology IRCCS, 20141 Milan, Italy

**Keywords:** concomitant chemoradiotherapy, organ preservation, trimodality, urinary bladder cancer

## Abstract

The aim of this study is to access the feasibility, toxicity profile, and tumour outcome of an organ preservation curative approach in non-metastatic muscle-invasive bladder cancer.

A retrospective analysis was conducted on patients affected by M0 bladder cancer, who refused cystectomy and were treated with a curative approach. The standard bladder preservation scheme included maximal transurethral resection of bladder tumour (TURBT) and combination of radiotherapy and platin-based chemotherapy, followed by endoscopic evaluation, urine cytology, and instrumental evaluation.

Thirteen patients fulfilled the inclusion criteria. TNM stage was cT2cN0M0 and cT2cNxM0, in 12 and one patients, respectively. All patients had transitional cell cancer. Twelve patients completed the whole therapeutic programme (a bimodal treatment without chemotherapy for one patient). Median follow-up is 36 months. None of the patients developed severe urinary or intestinal acute toxicity. In 10 patients with a follow-up > 6 months, no cases of severe late toxicity were observed. Response evaluated in 12 patients included complete response and stable disease in 11 patients (92%), and one patient (8%), respectively. At the time of data analysis (March 2016), 10 patients (77%) are alive with no evidence of disease, two patients (15%) died for other reasons, and one patient has suspicious persistent local disease.

The trimodality approach, including maximal TURBT, radiotherapy, and chemotherapy for muscle-invasive bladder cancer, is well-tolerated and might be considered a valid and feasible option in fit patients who refuse radical cystectomy.

## Introduction

The standard treatment for muscle-invasive bladder cancer remains radical cystectomy, with removal of bladder, regional lymph nodes (extended lymph node dissection) and surrounding structures (uterus or prostate gland), with urinary diversion [1–2]. This procedure is still associated with complication rates of up to 30% and, moreover, urinary diversion has a great impact on long-term urinary, gastrointestinal, and sexual function, decreasing significantly the patient’s quality of life [3–4].

Two cohorts of patients could benefit from a conservative approach: patients with a limited disease who wish to avoid aggressive surgery and patients with severe comorbidities who are not candidates for radical cystectomy.

Single modality bladder-preservation treatment consisting of transurethral resection of bladder tumour (TURBT), chemotherapy, or radiotherapy yields inferior results in terms of local tumour control and long-term survival [5–6].

No randomised studies comparing radical cystectomy and the bladder-sparing approach have been completed: the SPARE (Selective bladder Preservation Against Radical Excision) trial which expected to randomly compare radical cystectomy versus bladder preservation has been closed due to poor accrual [7]. Several prospective studies has been conducted over the past two decades showing survival rates of a trimodality approach, comparable to those of radical cystectomy or neoadjuvant chemotherapy followed by cystectomy series [8–11]. This combined treatment is based on maximal TURBT, followed by radiotherapy and concurrent chemotherapy.

Long-term data, recently published by the Radiation Therapy Oncology Group (RTOG), are important in establishing selective bladder-preserving treatments as a safe and effective alternative to cystectomy [12].

Consequently, bladder preservation is now considered as an option by the guidelines of the major scientific societies (National Comprehensive Cancer Network, European Association of Urology EAU) for selected patients [13–14]. However, our recent survey showed that bladder preservation is rarely used in Lombardy (Milan area), despite the availability of the latest radiotherapy technologies and the presence of a urology tumour board in at least half of the centres [15]. Our purpose, at the European Institute of Oncology, Milan, was to retrospectively analyse the feasibility, toxicity profile, and tumour control of the trimodality approach in non-metastatic muscle-invasive bladder cancer.

## Material and methods

The inclusion criteria in this retrospective study were as follows: (1) non-metastatic bladder cancer patients who refused cystectomy or were unfit for surgery; (2) patients treated with a trimodality approach, based on a combination of maximal TURBT, radiotherapy, and chemotherapy, with a curative intent; (3) written consensus for the treatment; (4) consent for the use of the data for educational and research purposes.

We retrospectively reviewed the medical records, treatments charts, radiotherapy plans, and follow-up appointments. In cases of missing follow-up data, the patients or their family doctors were contacted.

A radiological total-body evaluation was performed at the time of diagnosis to exclude other sites of disease. The approach was discussed, in all cases, by the institutional multidisciplinary tumour board. After the first TURBT showing muscle-invasive bladder cancer (> T2), the trimodality regimen was undertaken including the second maximal TURBT followed by radiation therapy + concomitant chemotherapy and evaluation (urine cytology, endoscopic evaluation, and radiological examination).

At the conclusion of the whole treatment, all patients were radiologically evaluated and, in cases of no evidence of disease, follow-up was undertaken.

Evaluation of acute and late toxicity was performed according to Common Terminology Criteria for Adverse Events (CTCAE v4.0) and the Radiation Therapy Oncology Group/European Organisation for Research and Treatment of Cancer (RTOG/EORTC) scoring systems [16–17].

## Results

Thirteen patients met the criteria of our study (five females and eight males, median age 65 years at the time of diagnosis) (Table 1). All patients were treated in our Institute, between June 2006 and January 2016.

The initial stage, according to the TNM stage system, was defined as cT2 cN0 for 12 patients, cT2 cNx for one patient with two suspicious pelvic enlarged nodes (about 1 cm diameter) on the staging abdominal CT with contrast. Non-metastatic disease was confirmed in all patients. Histology included transitional cell cancer in all cases.

In 10 patients, bladder preservation was administered for patients’ choice. In the other three patients, the conservative programme was chosen for clinical reasons.

Eleven patients completed the trimodality programme, including maximal TURBT followed by external radiation therapy and concomitant chemotherapy. One patient, affected by an important psychiatric disease, did not finish the whole programme, and we considered the treatment ended at the 24^th^ fraction of radiotherapy.

In one patient with cardiac disorders (arrhythmia requiring pace-maker) no chemotherapy was administered during radiotherapy.

**Figure 1.**
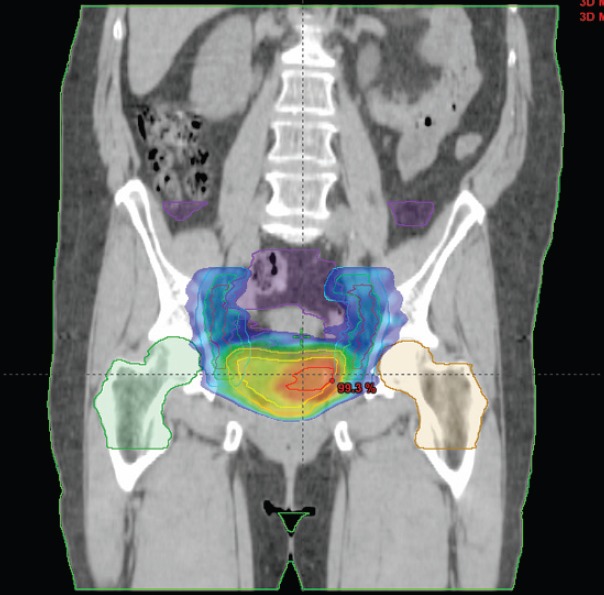
Dose distribution and volumes of IG-IMRT plan with the concomitant irradiation of lymph nodes of the small pelvis, whole bladder and a simultaneous concomitant boost to the site of the bladder tumour.

The external radiotherapy included the whole pelvis. In the initial study period, two patients were treated with three-dimensional (3D) conformal external beam radiotherapy through a four-field box technique. The following 11 patients underwent image-guided intensity-modulated radiotherapy (IG-IMRT), all with daily fractions in five consecutive days each week (Figure 1).

The treatment volumes included the bladder (with a median dose of 56 Gy), pelvic lymph nodes (with a median dose of 49 Gy) and a boost on the site of previous tumour (with a median dose of 62 Gy), determined by computed tomography (CT) scan, clinical information (pre-TURBT data, TURBT report, etc.) and magnetic resonance imaging (MRI), if available. IG-IMRT was performed on the outpatient basis.

As radiosensitising chemotherapy was employed weekly cisplatin (20 mg/m^2^/d, one day per week) or carboplatin (area under curve, one day per week) in nine and three patients, respectively. Carboplatin was used in patients with renal insufficiency or with another comorbidity contraindicating cisplatin use.

**Figure 2.**
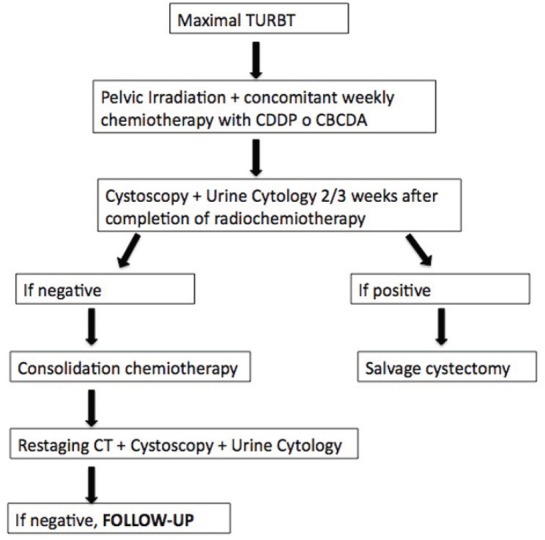
Schematic representation of the decision–making process in patients treated with bladder-sparing approach.

Six patients received adjuvant chemotherapy with cisplatin and gemcitabine for two or three courses, following the therapeutic scheme showed in Figure 2.

Considering the recent review published by Ploussard *et al*., illustrating not univocal data about the benefit of adjuvant chemotherapy [18], and according to EAU Guidelines [14], the following six patients received only chemotherapy concomitant to radiotherapy.

Two months after the completion of chemoradiotherapy, endoscopic evaluation (cystoscopy or TURBT), and urine cytology were performed.

During concomitant chemoradiotherapy, none of the patients developed acute G3–G4 urinary or gastroenteric toxicity. We observed neither an increase in grade 3–4 haematological toxicity and neuropathy with concurrent chemotherapy nor a decrease in treatment completion rates caused by cumulative toxicity.

One patient, affected concomitantly by multiple myeloma, showed a G3 haematological event and interrupted adjuvant chemotherapy after one course to receive support therapy. During adjuvant chemotherapy, one patient experienced a pulmonary embolism and one patient renal failure treated with adequate symptomatic therapy.

In 10 patients with a follow-up longer than 6 months, no cases of G3–G4 genitourinary and gastrointestinal toxicity were reported. The median follow-up is 36 months (range 2–101 months).

Twelve patients were evaluable for clinical response. Complete tumour response at the first follow-up, considered as the negativity of endoscopic examination and urinary cytology, was observed in 11 patients (92%); persistent bladder disease in one patient (8%), evidenced at the first endoscopic evaluation. Progressive disease was observed in none of the patients. One patient developed, after 7 months, an *in situ* bladder recurrence and was treated with BCG (bacillus Calmette-Guérin) instillations. One patient experienced a local invasive bladder relapse, 9 months after the end of the trimodality treatment, and, consequently, underwent successful salvage cystectomy.

At the time of data analysis (March 2016), the status can be defined in all patients. Out of 13 patients, 10 patients (77%) are alive without evidence of disease, nine of them (69%) retain an intact bladder; one patient, with a suspect of persistent bladder tumour, is waiting for diagnostic TURBT; two patients (15%) died, one for cardiovascular event (the same patient who interrupted chemoradiotherapy for psychiatric disease), and one for multiple myeloma.

Both of these two death cases are part of the three patients in which the conservative treatment was chosen for important comorbidities. The results observed in this patient subgroup were determined by underlying disease, even if the patient, who underwent bimodal treatment without chemotherapy, is still alive without evidence of disease after 7 months of follow-up.

The patient with cNx stage at diagnosis is, at the time of data analysis, alive retaining an intact bladder and without evidence of disease after a follow-up of more than 8 years.

## Discussion

Our single-institutional study showed that bladder preservation approach can allow the maintenance of a well-functioning organ in a high percentage of selected patients with MIBC. Indeed, 69% of all our patients are alive with no evidence of cancer and with intact well-functioning urinary bladder at median follow-up of 37 months. These figures are similar to those observed in large surgical series and other bladder preservation reports [8–9].

We are aware of numerous drawbacks of our series including, apart from the fact it was a unicenter study, low number of patients, long accrual time, and retrospective character. However, to the best of our knowledge, this is one of the first reports on bladder preservation from Lombardy showing feasibility and good results, in particular, in fit patients (8 out of 10 fit patients, i.e., 80%, remain alive with intact bladder). We do believe our experience will encourage other multidisciplinary teams to consider bladder preservation in selected fit patients [15].

The long accrual time observed in our study reflects the common issue of the treatment choice in muscle-invasive bladder cancer. This is probably due to the poor spread of conservative treatments for invasive bladder cancer in our clinical practice despite numerous recent reviews showing encouraging results of trimodality therapy in selected patients [19–22].

In the absence of randomised trials, it is difficult to compare bladder preservation with standard cystectomy, because of the wide heterogeneity in patient selection. Interestingly, a recent systematic review of clinical retrospective and prospective trials including 13,396 patients, showed the median five-year overall survival rate of 57% in the trimodality group, when compared with 52% (*p* = 0.04), 51% (*p* = 0.02), and 53% (*p* = 0.38) in the whole group receiving radical cystectomy or the group treated with radical cystectomy alone or radical cystectomy+chemotherapy, respectively [10]. Indeed, some investigators ask the provocative question on the organ-sparing multimodality treatment for muscle-invasive bladder cancer: can we continue to ignore the evidence? [23].

The ideal candidate for bladder preservation are fit patients with a small tumour (less than 5 cm), unifocal, no microscopic residual after TURBT, the absence of ureteral obstruction or hydronephrosis, no association with *in situ* carcinoma (accurately diagnosed by biopsies of suspicious areas and analysis of histology of previous TURBT) no evidence of pelvic lymph node disease [24–25]. A recent review published by Smith *et al*. shows that 10–15% of medically operable patients are good candidates for bladder preservation [21]. In our series, all patients met the criteria for the conservative treatment as alternative to radical cystectomy.

The toxicity of trimodality approach, employed in our study, was acceptable with no direct radiotherapy-related severe events. Except in studies using neoadjuvant or adjuvant chemotherapy, where toxicity seems higher, this rate ranged from 10% to 36%, while the majority (80–90%) of patients did complete the entire course of treatment. The main toxicities are haematological, gastrointestinal, and genitourinary. Neuropathy may be reported in cases of cisplatin-based concurrent chemotherapy. The BC2001 trial reported neither an increase in G3–G4 toxicity with concurrent chemotherapy compared with radiotherapy alone nor a decrease in radiotherapy completion rates caused by toxicity [25].

In the earlier literature series, cystectomy for contracted bladder was necessary in up to 2% of patients receiving a conservative approach [21]. In our patients, the complications have been observed predominantly during adjuvant chemotherapy (G3 myelotoxicity, renal failure, and lung thromboembolism), confirming higher toxicity of concomitant and adjuvant chemotherapy in respect of concomitant only chemotherapy in bladder cancer preservation; this finding confirms the fragile general conditions of the bladder cancer patients. Indeed, the most recent bladder preservation reports claim that TURBT followed by concomitant chemoradiotherapy (with no adjuvant or neoadjuvant chemotherapy) may be considered the optimal approach in terms of tolerability and efficacy [26].

From our limited experience, we learnt two essential assumptions: first, patients’ good compliance is fundamental during the active treatment and subsequently, because of the need both of prompt salvage cystectomy in cases of no tumour response or local failure, and of lifelong surveillance; second, in such an assessment, a close and continuous collaboration between Urologists, Medical Oncologists and Radiation Oncologists becomes of extreme importance, in order to reach an optimal multidisciplinary approach. In Lombardy, a prospective observational study on the bladder preservation has been just undertaken reinforcing uro-oncology teamwork.

## Conclusion

In conclusion, the trimodality approach including maximal TURBT, radiotherapy, and chemotherapy for muscle-invasive bladder cancer, in patients who refuse radical cystectomy or are unfit for surgery, could be considered as a good and feasible therapeutic option and results in a low rate of acute and late toxicity.

Long-term follow-up and more prospective multicentre research are needed to confirm the results of our retrospective study.

**Abbreviations used:** TURBT, transurethral bladder tumour resection; G, grade; Gy, Grey, IG-IMRT, image-guided intensity-modulated radiotherapy, CT, computed tomography; RT, radiation therapy.

## Figures and Tables

**Table 1.** Patient, tumour, and maximum toxicity evaluated during chemoradiotherapy.

Patients	*N* patients (tot 13)	Percentage
**Patient characteristics**		
*Age (years)* Mean Range *Gender* Male Female *Smoker* Yes No Unknown	65 42–80 8 5 8 (4 ex-smoker) 2 3	61.5% 38.5% 61.5% 15.4% 23%
**Tumour characteristics**		
*Histology* Transitional cell cancer *Primary tumour stage* T2 T3 T4 *Regional lymph node stage* N0 Nx *Hydronephrosis* Yes No	13 13 – – 12 1 1 12	100% 100% – – 92% 8% 8% 92%
**Toxicity** **RTOG/EORTC scale (15)**		
*Genitourinary* Grade 0 Grade 1 Grade 2 Grade 3 *Gastrointestinal* Grade 0 Grade 1	2 7 4 – 5 8	15% 54% 31% – 61.5% 38.5%
**Toxicity** **CTCAE v4.0 (14)**		
*Urinary* Grade 0 Grade 1 Grade 2 *Gastrointestinal* Grade 0 Grade 1 *Anaemia* Grade 0 Grade 1 Grade 3 *Asthenia* Grade 0 Grade 1 Grade 2 *Leucopenia* Grade 0 Grade 1 Grade 2 *Thrombocytopenia* Grade 0 Grade 1 *Nausea* Grade 0 Grade 1 *Pelvic pain* Grade 0 Grade 2 *Alopecia* Grade 0 Grade	2 6 5 7 6 10 2 1 7 4 2 10 2 1 12 1 8 5 11 2 12 1	15.4% 46.1% 38.5% 54% 46% 77% 15.4% 7.6% 53.6% 31% 15.4% 77% 15.4% 7.6% 92% 8% 61.5% 38.5% 84.6% 15.4% 92% 8%
